# Quantifying SARS-CoV-2 transmission suggests epidemic control with digital contact tracing

**DOI:** 10.1126/science.abb6936

**Published:** 2020-03-31

**Authors:** Luca Ferretti, Chris Wymant, Michelle Kendall, Lele Zhao, Anel Nurtay, Lucie Abeler-Dörner, Michael Parker, David Bonsall, Christophe Fraser

**Affiliations:** 1 Big Data Institute, Li Ka Shing Centre for Health Information and Discovery, University of Oxford, Oxford, UK.; 2 Wellcome Centre for Ethics and the Humanities and Ethox Centre, University of Oxford, Oxford, UK.; 3 Oxford University NHS Trust, University of Oxford, Oxford, UK.; 4 Wellcome Centre for Human Genetics, University of Oxford, Oxford, UK.

## Abstract

The newly emergent human virus SARS-CoV-2 (severe acute respiratory syndrome–coronavirus 2) is resulting in high fatality rates and incapacitated health systems. Preventing further transmission is a priority. We analyzed key parameters of epidemic spread to estimate the contribution of different transmission routes and determine requirements for case isolation and contact tracing needed to stop the epidemic. Although SARS-CoV-2 is spreading too fast to be contained by manual contact tracing, it could be controlled if this process were faster, more efficient, and happened at scale. A contact-tracing app that builds a memory of proximity contacts and immediately notifies contacts of positive cases can achieve epidemic control if used by enough people. By targeting recommendations to only those at risk, epidemics could be contained without resorting to mass quarantines (“lockdowns”) that are harmful to society. We discuss the ethical requirements for an intervention of this kind.

## Instantaneous contact tracing

New analyses indicate that severe acute respiratory syndrome–coronavirus 2 (SARS-CoV-2) is more infectious and less virulent than the earlier SARS-CoV-1, which emerged in China in 2002. Unfortunately, the current virus has greater epidemic potential because it is difficult to trace mild or presymptomatic infections. As no treatment is currently available, the only tools that we can currently deploy to stop the epidemic are contact tracing, social distancing, and quarantine, all of which are slow to implement. However imperfect the data, the current global emergency requires more timely interventions. Ferretti *et al.* explored the feasibility of protecting the population (that is, achieving transmission below the basic reproduction number) using isolation coupled with classical contact tracing by questionnaires versus algorithmic instantaneous contact tracing assisted by a mobile phone application. For prevention, the crucial information is understanding the relative contributions of different routes of transmission. A phone app could show how finite resources must be divided between different intervention strategies for the most effective control.

*Science*, this issue p. eabb6936

## Abstract (structured)

**INTRODUCTION:**

Coronavirus disease 2019 (COVID-19), caused by severe acute respiratory syndrome–coronavirus 2 (SARS-CoV-2), has clear potential for a long-lasting global pandemic, high fatality rates, and incapacitated health systems. Until vaccines are widely available, the only available infection prevention approaches are case isolation, contact tracing and quarantine, physical distancing, decontamination, and hygiene measures. To implement the right measures at the right time, it is of crucial importance to understand the routes and timings of transmission.

**RATIONALE:**

We used key parameters of epidemic spread to estimate the contribution of different transmission routes with a renewal equation formulation, and analytically determined the speed and scale for effective identification and contact tracing required to stop the epidemic.

**RESULTS:**

We developed a mathematical model for infectiousness to estimate the basic reproductive number R_0_ and to quantify the contribution of different transmission routes. To parameterize the model, we analyzed 40 well-characterized source-recipient pairs and estimated the distribution of generation times (time from infection to onward transmission). The distribution had a median of 5.0 days and standard deviation of 1.9 days. We used published parameters for the incubation time distribution (median 5.2 days) and the epidemic doubling time (5.0 days) from the early epidemic data in China.

The model estimated R_0_ = 2.0 in the early stages of the epidemic in China. The contributions to R_0_ included 46% from presymptomatic individuals (before showing symptoms), 38% from symptomatic individuals, 10% from asymptomatic individuals (who never show symptoms), and 6% from environmentally mediated transmission via contamination. Results on the last two routes are speculative. According to these estimates, presymptomatic transmissions alone are almost sufficient to sustain epidemic growth.

To estimate the requirements for successful contact tracing, we determined the combination of two key parameters needed to reduce R_0_ to less than 1: the proportion of cases who need to be isolated, and the proportion of their contacts who need to be quarantined. For a 3-day delay in notification assumed for manual contact tracing, no parameter combination leads to epidemic control. Immediate notification through a contact-tracing mobile phone app could, however, be sufficient to stop the epidemic if used by a sufficiently high proportion of the population.

We propose an app, based on existing technology, that allows instant contact tracing. Proximity events between two phones running the app are recorded. Upon an individual’s COVID-19 diagnosis, contacts are instantly, automatically, and anonymously notified of their risk and asked to self-isolate. Practical and logistical factors (e.g., uptake, coverage, R_0_ in a given population) will determine whether an app is sufficient to control viral spread on its own, or whether additional measures to reduce R_0_ (e.g., physical distancing) are required. The performance of the app in scenarios with higher values of R_0_ can be explored at https://bdi-pathogens.shinyapps.io/covid-19-transmission-routes/.

**CONCLUSION:**

Given the infectiousness of SARS-CoV-2 and the high proportion of transmissions from presymptomatic individuals, controlling the epidemic by manual contact tracing is infeasible. The use of a contact-tracing app that builds a memory of proximity contacts and immediately notifies contacts of positive cases would be sufficient to stop the epidemic if used by enough people, in particular when combined with other measures such as physical distancing. An intervention of this kind raises ethical questions regarding access, transparency, the protection and use of personal data, and the sharing of knowledge with other countries. Careful oversight by an inclusive advisory body is required.

## Teaser

Instantaneous contact tracing and notifications by mobile phone app could potentially stop the COVID-19 epidemic.

## Graphical abstract

**Figure:**
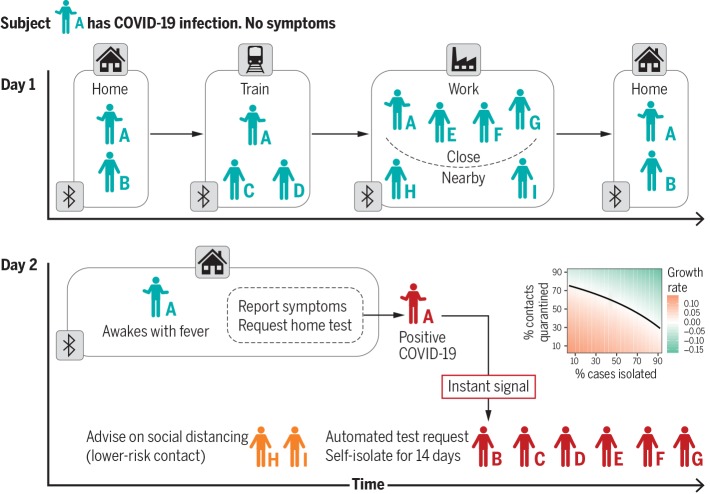
Instant contact tracing can reduce the proportion of cases that need to be isolated and contacts who need to be quarantined to achieve control of an epidemic. Subject A becomes symptomatic after having had contact with other people in different settings the day before. Contacts are notified and quarantined where needed. In the inset, the green area indicates the success rates needed to control an epidemic with R_0_ = 2 (i.e., negative growth rates after isolating cases and quarantining their contacts).

Coronavirus disease 2019 (COVID-19) is a rapidly spreading infectious disease caused by severe acute respiratory syndrome–coronavirus 2 (SARS-CoV-2), a betacoronavirus, which has now established a global pandemic. Around half of infected individuals become reported cases, and with intensive care support, the case fatality rate is approximately 2% (*1*). More concerning is that the proportion of cases requiring intensive care support is 5%, and patient management is complicated by requirements to use personal protective equipment and engage in complex decontamination procedures (*2*). Fatality rates are likely to be higher in populations older than in Hubei province (such as in Europe) and in low-income settings where critical care facilities are lacking (*3*). The public health cost of failing to achieve sustained epidemic suppression has been estimated as 250,000 lives lost in the next few months in Great Britain, and 1.1 to 1.2 million in the United States, even with the strongest possible mitigation action to “flatten the curve” (*4*). Even modest outbreaks will see fatality rates climb as hospital capacity is overwhelmed, and the indirect effects caused by compromised health care services have yet to be quantified.

No treatment is currently available, and vaccines are not expected to be sufficiently widely available to control the epidemic within the coming year. The only approaches that we currently have available to stop the epidemic are those of classical epidemic control, such as case isolation, contact tracing and quarantine, physical distancing, and hygiene measures.

The basic reproduction number R_0_ is the typical number of infections caused by an individual in the absence of widespread immunity. Once immunity becomes widespread, the effective reproduction number R will become lower than R_0_; once R is less than 1, the population has herd immunity and the epidemic declines. Immunity can only safely be obtained by vaccination. Here we use the term “sustained epidemic suppression” to mean a reduction of R to less than 1 by changing nonimmunological conditions of the population that affect transmission, such as social contact patterns.

The biological details of transmission of betacoronaviruses are known in general terms: These viruses can pass from one individual to another through exhaled droplets (*5*), aerosol (*6*), contamination of surfaces (*7*), and possibly through fecal-oral contamination (*8*). Here, we compare different transmission routes that are more closely aligned to their implications for prevention. Specifically, we propose four categories:

1) Symptomatic transmission: direct transmission from a symptomatic individual, through a contact that can be readily recalled by the recipient.

2) Presymptomatic transmission: direct transmission from an individual that occurs before the source individual experiences noticeable symptoms. (Note that this definition may be context-specific—for example, based on whether it is the source or the recipient who is asked whether the symptoms were noticeable.)

3) Asymptomatic transmission: direct transmission from individuals who never experience noticeable symptoms. This can only be established by follow-up, as single–time point observation cannot fully distinguish asymptomatic from presymptomatic individuals.

4) Environmental transmission: transmission via contamination, and specifically in a way that would not typically be attributable to contact with the source in a contact survey (i.e., this does not include transmission pairs who were in extended close contact, but for whom in reality the infectious dose passed via the environment instead of more directly). These could be identified in an analysis of spatial movements.

We acknowledge that boundaries between these categories may be blurred, but defined broadly these categories have different implications for prevention, responding differently to classical measures of case isolation and quarantining contacts (*9*, *10*) [for a specific application to COVID-19, see below (*11*)].

Evidence exists for each of these routes of transmission: symptomatic (*12*), presymptomatic (*13*), asymptomatic (*14*), and environmental (*12*). For prevention, the crucial information is the relative frequency of different routes of transmission so as to allocate finite resources between different intervention strategies.

Li *et al*. (*12*) presented self-reported data on exposure for the first 425 cases in Wuhan; some of these recorded visits to the Huanan Seafood Wholesale Market. The generalizability of transmission in that setting to other settings is highly uncertain, as this large-scale event seeded the epidemic in the absence of any knowledge about the disease. After closure of the Huanan Seafood Wholesale Market on 1 January 2020, of 240 cases with no exposure to any wet market, 200 individuals (83%) reported no exposure to an individual with respiratory symptoms. Inaccurate recall may explain some responses, including failing to notice symptoms that were exceptional at a time before awareness of the disease began, but it is unlikely to be as much as 83% of them, implying that many individuals were infected by nonsymptomatic individuals.

The situation in Singapore at first glance appears different, because unlike in Wuhan, many individuals were linked to an identified symptomatic source. However, the main difference is that the linkage was retrospective, such that linkage could be established even if transmission occurred before a case was symptomatic. As of 5 March 2020, there were 117 cases, of which 25 were imported. By devoting considerable resources, including police investigation, 75 of the 92 cases of local transmission were traced back to their presumed exposure, either to a known case or to a location linked to spread (*15*). Linking cases via a location generally includes the possibility of environmentally mediated transmission. Therefore, the large fraction of traceable transmission in Singapore does not contradict the large fraction without symptomatic exposure in Wuhan. However, it does suggest that transmission from asymptomatic, rather than presymptomatic, individuals is not a major driver of spread. Although serological surveys are currently lacking, other lines of evidence suggest that the scenario of many asymptomatic infections for each symptomatic one is unlikely. Testing of 1286 close contacts of confirmed cases found that among 98 individuals testing positive, only 20% did not have symptoms at first clinical assessment (*16*). Among 634 individuals testing positive onboard the Diamond Princess cruise ship, the proportion of individuals without symptoms was found to be 52%; the proportion who were asymptomatic (rather than presymptomatic) was estimated as 18% (*17*). Testing of passengers onboard six repatriation flights from Wuhan suggests that 40 to 50% of infections were not identified as cases (*4*, *18*). Viral loads of mild cases have been found to be less than those of severe cases by a factor of 60 (*19*), and it is likely that the viral loads of asymptomatic individuals are lower still, with possible implications for infectiousness and diagnosis.

The most accurate and robust quantification of the relative frequency of routes of transmission would be a well-designed prospective cohort study with detailed journal and phylogenetic investigations. However, the current global emergency requires timely estimates using imperfect data sources. We performed a detailed analysis of the timing of events in defined transmission pairs, derived the generation time distribution, and attributed a probability for each pair that transmission was presymptomatic. We also fit a mathematical model of infectiousness through the four routes discussed above over the course of infection. This allowed us to calculate R_0_, estimate the proportion of transmission from different routes, and make predictions about whether contact tracing and isolation of known cases would be enough to prevent spread of the epidemic.

## Estimating SARS-CoV-2 transmission parameters

We used the exponential growth rate of the epidemic, *r*, from the early stages of the epidemic in China, such that the effect of control measures discussed later will be relative to the early stages of an outbreak, exemplified by baseline contact patterns and environmental conditions in Hubei during that period. We note that this assumption is implicit in many estimates of R_0_. The epidemic doubling time *T*_2_ is equal to log_e_(2)/*r.* We used the value *r* = 0.14 per day (*20*), corresponding to a doubling time of 5.0 days.

**Fig. 1:**
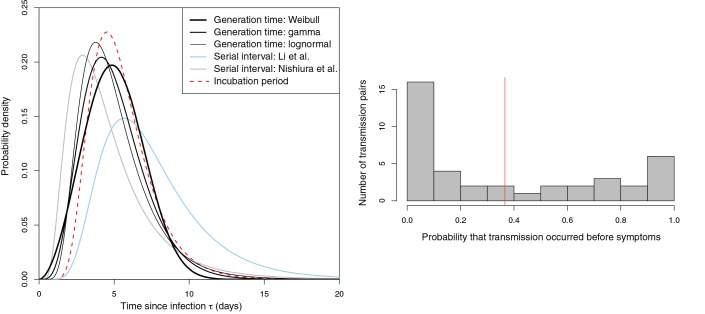
Quantifying transmission timing in 40 transmission pairs. Left: Our inferred generation time distributions, in black; thicker lines denote higher support for the corresponding functional form, with the Weibull distribution being the best fit. For comparison, we also include the serial interval distributions previously reported by Li *et al*. (*12*) (light blue) and Nishiura* et al*. (*22*) (gray) and the incubation period distribution we used here, from Lauer *et al*. (*21*) (dashed red line). Right: Distribution of the posterior probability of presymptomatic transmission for each of the 40 transmission pairs. The red vertical line shows the mean probability.

The incubation period is defined as the time between infection and onset of symptoms. It is estimated as the time between exposure and report of noticeable symptoms. We used the incubation period distribution calculated in (*21*). The distribution is lognormal with a mean of 5.5 days, a median of 5.2 days, and a standard deviation of 2.1 days, and is included with our results in Fig. 1.

The generation time is defined for source-recipient transmission pairs as the time between the infection of the source and the infection of the recipient. Because time of infection is generally not known, the generation time is often approximated by the serial interval, which is defined as the time between the onset of symptoms of the source and the onset of symptoms of the recipient. We did not take that approach here; instead, we directly estimated the generation time distribution from 40 source-recipient pairs. These pairs were manually selected according to high confidence of direct transmission inferred from publicly available sources at the time of writing (March 2020), and with known time of onset of symptoms for both source and recipient. We combined dates of symptom onset with intervals of exposure for both source and recipient (when available) and the above distribution of incubation times, and from these we inferred the distribution of generation times. The distribution is best described by a Weibull distribution (Akaike information criterion = 148.4, versus 149.9 for gamma and 152.3 for lognormal distribution) with mean and median equal to 5.0 days and standard deviation of 1.9 days, shown in the left panel of Fig. 1. We also show the results of a sensitivity analysis to different functional forms, as well as two previously published serial interval distributions (*12*,***22*). Uncertainty in the fit of the distribution is shown in fig. S1. Our distribution is robust with respect to the choice of transmission events (fig. S2). Correlation in the uncertainty between the inferred mean and standard deviation is shown in fig. S3. The distribution of serial intervals for these pairs is shown in fig. S4. The countries from which the transmission pair data were obtained are shown in fig. S5.

For each of the 40 transmission pairs, we estimated the posterior probability that transmission was presymptomatic (i.e., occurred before the onset of symptoms in the infector). We used a Bayesian approach with an uninformative prior (i.e., transmission before or after symptoms equally likely). The 40 probabilities inferred are shown in the right panel of Fig. 1; the mean probability is 37% [95% confidence interval (CI), 27.5 to 45%], which can be interpreted as the fraction of presymptomatic transmission events out of presymptomatic plus symptomatic transmission events. This mean probability over all pairs is close to our prior, but the bimodal distribution of individual probabilities in Fig. 1 shows that these are typically far from the prior (i.e., the data are strongly informative). Uncertainty in the value of this fraction is shown in fig. S6. The value does not depend significantly on the choice of prior (figs. S7 and S8), on the functional form of the distribution of generation times (figs. S9 and S10), or on the choice of transmission events (fig. S11).

## A general mathematical model of SARS-CoV-2 infectiousness

We used a mathematical formalism (*23*) that describes how infectiousness varies as a function of time since infection, τ, for a representative cohort of infected individuals. This includes heterogeneity between individuals, and averages over those individuals who infect few others and those who infect many. This average defines the function β(τ). Infectiousness may change with τ as a result of changing disease biology (notably viral shedding) and/or changing contact with others. The area under the β curve is the reproduction number R_0_.

We decomposed β(τ) into four contributions that reflect our categorization above, namely asymptomatic transmission, presymptomatic transmission, symptomatic transmission, and environmental transmission. The area under the curve of one of these contributions gives the mean total number of transmissions over one full infection, via that route—asymptomatic, presymptomatic, symptomatic, or environmental—which we define to be R_A_, R_P_, R_S_, and R_E_, respectively. The sum of these is R_0_.

**Fig. 2:**
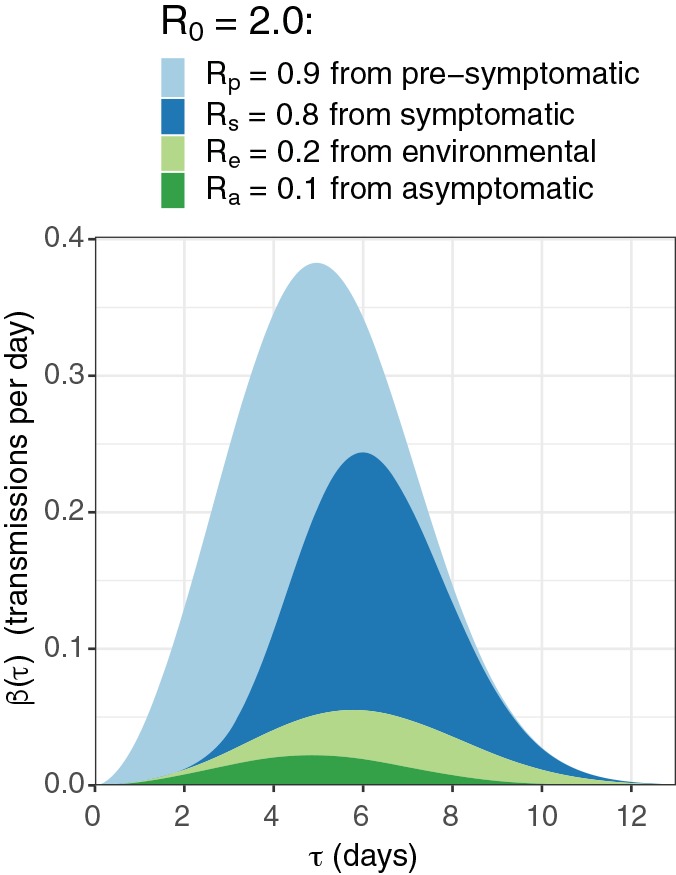
Our model of infectiousness. The average infectiousness (rate of infecting others), β, is shown as a function of the amount of time since infection, τ. The total colored area found between two values of τ is the number of transmissions expected in that time window. The total colored area over all values of τ is the number of transmissions expected over the full course of one infection (i.e., the basic reproduction number R_0_). The different colors indicate the contributions of the four routes of transmission, so that the total area of one color over all values of τ is the average number of transmissions via that route over the whole course of infection: R_P_, R_S_, R_E_, and R_A_ for presymptomatic, symptomatic, environmentally mediated, and asymptomatic transmission, respectively. Note that the colors are stacked on top of one another (i.e., the lower colors are not in front, and the higher colors are not behind and partially obscured). Values are rounded to one decimal place. Stopping the spread of disease requires reduction of R to less than 1: blocking transmission, from whatever combination of colors and values of τ we can achieve, such that the total area is halved.

The mathematical form for β(τ) is

$$\beta \left(\tau \right)=\underset{\mathrm{asymptomatic}}{\underset{︸}{P_{a}x_{a}\beta_{s}\left(\tau \right)}}+\underset{\mathrm{presymptomatic}}{\underset{︸}{\left(1-P_{a}\right)\left[1-s\left(\tau \right)\right]\beta_{s}\left(\tau \right)}}+\underset{\mathrm{symptomatic}}{\underset{︸}{\left(1-P_{a}\right)s\left(\tau \right)\beta_{s}\left(\tau \right)}}+\underset{\mathrm{environmental}}{\underset{︸}{\int_{l=0}^{\tau }\beta_{s}\left(\tau -l\right)E\left(l\right)\;dl}}$$

where β*_s_*(τ) is the infectiousness of an individual currently either symptomatic or presymptomatic, at age of infection τ. Other parameters in this expression, and those feeding into it indirectly, are listed in Table 1. A detailed discussion of this expression, including its assumptions, is found in the supplementary materials. The priors chosen for parameters not directly calculated from data are shown in fig. S12. The infectiousness model result using central values of all parameters is shown in Fig. 2.

**Table 1:** Parameters of the infectiousness model.

**Name**	**Symbol**	**Description**	**Central value**	**Uncertainty**	**Source**
*Parameters directly calculated from data*					
Doubling time	*T*_2_	The time taken for the epidemic to double in size during the early uncontrolled phase of expansion	5.0 days	95% CI: 4.2–6.4	(*20*)
Incubation period (two parameters)	*s*(τ)	Lognormal meanlog Lognormal sdlog	1.644 0.363	95% CI: 1.495–1.798 95% CI: 0.201–0.521	(*21*)
Generation time (two parameters)	*w*(τ)	Weibull shape Weibull scale	2.826 5.665	95% CI: 1.75–4.7 95% CI: 4.7–6.9	This paper
*Parameters with Bayesian priors informed by anecdotal reports or indirect evidence*					
Proportion asymptomatic	*P*_a_	The proportion of infected individuals who are asymptomatic	0.4	Prior = beta (α = 1.5, β = 1.75) Mode = 0.4 Mean = 0.46	Media reports (Diamond Princess)
Relative infectiousness of asymptomatics	*x*_a_	The ratio of infectiousness of asymptomatic individuals to infectiousness of symptomatic individuals	0.1	Prior = beta (α = 1.5, β = 5.5) Mode = 0.1 Mean = 0.21	Observation of few missing links in Singapore outbreak to date [suggestion from (*19*)]
Fraction of all transmission that is environmentally mediated	R_E_/R_0_	Self-explanatory	0.1	Prior = beta (α = 1.5, β = 5.5) Mode = 0.1 Mean = 0.21	Anecdotal observation that many infections can be traced to close contacts once detailed tracing is completed
Environmental infectiousness	*E*(*l*)	Rate at which a contaminated environment infects new people after a time lag *l*	3	Box function (0, *n*) days, prior for *n* = gamma (shape = 4, rate = 1) Mode = 3 Mean = 4	(*39*); variety of values for many different surfaces

By drawing input parameter sets from the uncertainties shown in Table 1, we quantified our uncertainty in R_0_ and its four contributions. The resulting values are shown in Table 2, and their underlying distributions are shown in fig. S13. Two-dimensional distributions showing correlations in uncertainty are shown in fig. S14. The estimate of R_P_/(R_P_ + R_S_) obtained by this method is 0.55 (CI, 0.37 to 0.72), which is larger than the estimate of 0.37 (CI, 0.28 to 0.45) from our analysis of the 40 transmission pairs but with overlapping uncertainty.

**Table 2:** R_0_ and its components.

	**Presymptomatic**	**Symptomatic**	**Environmental**	**Asymptomatic**	**Total R_0_**
Absolute	Point estimate: 0.9 Uncertainty median: 0.7 CI: 0.2–1.1	Point estimate: 0.8 Uncertainty median: 0.6 CI: 0.2–1.1	Point estimate: 0.2 Uncertainty median: 0.4 CI: 0.0–1.3	Point estimate: 0.1 Uncertainty median: 0.2 CI: 0.0–1.2	Point estimate: 2.0 Uncertainty median: 2.1 CI: 1.7–2.5
Fraction of R_0_	Point estimate: 0.47 Uncertainty median: 0.35 CI: 0.11–0.58	Point estimate: 0.38 Uncertainty median: 0.28 CI: 0.09–0.49	Point estimate: 0.1 by assumption Uncertainty median: 0.19 CI: 0.02–0.56	Point estimate: 0.06 Uncertainty median: 0.09 CI: 0.00–0.57	1 by definition

We define θ as the fraction of all transmissions that do not come from direct contact with a symptomatic individual: 1 – (*R*_S_/*R*_0_). This corresponds to the θ of (*9*) in the case where there is only presymptomatic and symptomatic transmission. From Table 2, this is 0.62 (CI, 0.50 to 0.92). The value of θ observed during an exponentially growing epidemic will be distorted when the timings of the different contributions to transmission occur at different stages of the infection, as a result of overrepresentation of recently infected individuals. This effect can be calculated through use of the renewal equation, as was recently done to calculate the distribution of time from onset of COVID-19 symptoms to recovery or death (*20*) (see supplementary materials). We calculated the θ that would be observed with the early exponential growth seen in China as 0.68 (CI, 0.56 to 0.92). The correction due to the epidemic dynamics is small relative to parameter uncertainties.

We developed our mathematical model of infectiousness into a web application where users can test the effect of alternative parameter combinations (*24*).

## Modeling case isolation and contract tracing with quarantine

We modeled the combined impact of two interventions: (i) isolating symptomatic individuals, and (ii) tracing the contacts of symptomatic cases and quarantining them. These interventions aim to stop the spread of the virus by reducing the number of transmissions from both symptomatic individuals and their contacts (who may not be symptomatic) while minimizing the impact on the larger population. In practice, neither intervention will be successful or possible for 100% of individuals. The success rate of these interventions determines the long-term evolution of the epidemic. If the success rates are high enough, the combination of isolation and contact tracing/quarantining could bring R below 1 and therefore effectively control the epidemic.

**Fig. 3:**
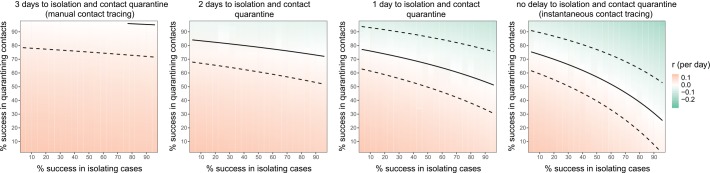
Quantifying intervention success. Heat map plot shows the exponential growth rate of the epidemic *r* as a function of the success rate of instant isolation of symptomatic cases (*x* axis) and the success rate of instant contact tracing (*y* axis). Positive values of *r* (red) imply a growing epidemic; negative values of *r* (green) imply a declining epidemic, with greater negative values implying faster decline. The solid black line shows *r *= 0 (i.e., the threshold for epidemic control). The dashed lines show uncertainty in the threshold due to uncertainty in R_0_ (see figs. S15 to S17). The different panels show variation in the delay associated with the intervention, from initiation of symptoms to case isolation and quarantine of contacts.

An analytical mathematical framework for the combined impact of these two interventions on an epidemic was previously derived in (*9*). In the supplementary materials, we solve these equations using our infectiousness model above—that is, quantifying how the SARS-CoV-2 epidemic is expected to be controlled (or not) by case isolation and the quarantining of traced contacts. Our results are shown in Fig. 3. The black line shows the threshold for epidemic control: Combined success rates in the region to the upper right of the black line are sufficient to reduce R to less than 1. The *x* axis is the success rate of case isolation, which can be thought of either as the fraction of symptomatic individuals isolated (assuming perfect prevention of transmission upon isolation) or the degree to which the infectiousness of symptomatic individuals is reduced (assuming all of them are isolated). The *y* axis is the success rate of contact tracing; similarly, this can be thought of as the fraction of all contacts traced (assuming perfectly successful quarantine upon tracing) or the degree to which infectiousness of contacts is reduced (assuming all of them are traced). These results for intervention effectiveness, and their dependence on all parameters in our combined analysis, can be explored through the same web interface as for our model of infectiousness (*24*).

Delays in these interventions make them ineffective at controlling the epidemic (Fig. 3): Traditional manual contact-tracing procedures are not fast enough for SARS-CoV-2. However, a delay between confirming a case and finding that person’s contacts is not inevitable. Specifically, this delay can be avoided by using a mobile phone app.

## Epidemic control with instant digital contact tracing

A mobile phone app can make contact tracing and notification instantaneous upon case confirmation. By keeping a temporary record of proximity events between individuals, it can immediately alert recent close contacts of diagnosed cases and prompt them to self-isolate.

Apps with similar aims have been deployed in China. Public health policy was implemented using an app that was not compulsory but was required to move between quarters and into public spaces and public transport. The app allows a central database to collect data on user movement and coronavirus diagnosis and displays a green, amber, or red code to relax or enforce restrictions on movement. The database is reported to be analyzed by an artificial intelligence algorithm that issues the color codes (*25*). The app is a plug-in for the WeChat and Alipay apps and has been generally adopted.

Mainland China outside of Hubei province received substantially more introductions from Wuhan than did any other locality, following mass movements of people around the Chinese New Year and the start of the Wuhan lockdown (*26*). Despite this, sustained epidemic suppression has been achieved in China: Fewer than 150 new cases have been reported each day from 2 March to 22 April, down from thousands each day at the height of the epidemic. South Korea has also achieved sustained epidemic suppression—8 new cases on 23 April, down from a peak of 909 on 29 February—and is also using a mobile phone app for recommending quarantine. Both the Chinese and South Korean apps have come under public scrutiny over issues of data protection and privacy.

**Fig. 4:**
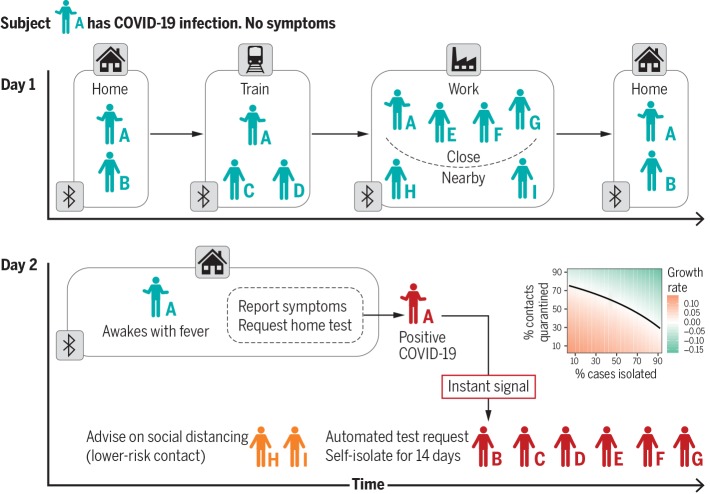
A schematic of app-based COVID-19 contact tracing. Contacts of individual A (and all individuals using the app) are traced using low-energy Bluetooth connections with other app users. Individual A requests a SARS-CoV-2 test (using the app) and that person’s positive test result triggers an instant notification to individuals who have been in close contact. The app advises isolation for the case (individual A) and quarantine of the individual’s contacts.

With our result in Fig. 3 implying the need for extremely rapid contact tracing, we set out to design a simple and widely acceptable algorithm from epidemiological first principles, using common smartphone functionality. The method is shown in Fig. 4. The core functionality is to replace a week’s work of manual contact tracing with instantaneous signals transmitted to and from a central server. Coronavirus diagnoses are communicated to the server, enabling recommendation of risk-stratified quarantine and physical distancing measures in those now known to be possible contacts, while preserving the anonymity of the infected individual. Tests could be requested by symptomatic individuals through the app.

The simple algorithm can easily be refined to be more informative—for example, quarantining areas if local epidemics become uncontrolled, quarantining whole households, or performing second- or third-degree contact tracing if case numbers are rising, which would result in more people being preemptively quarantined. Algorithmic recommendations can also be manually overridden where public health officials gather more specific evidence. The app can serve as the central hub of access to all COVID-19 health services, information, and instructions, and as a mechanism to request food or medicine deliveries during self-isolation.

In the context of a mobile phone app, Fig. 3 paints an optimistic picture. There is no delay between case confirmation and notification of contacts; thus, the delay for the contact quarantine process is the period from an individual experiencing symptoms to their contacts entering quarantine. The delay between symptom development and case confirmation will decrease with faster testing protocols, and indeed could become instant if presumptive diagnosis of COVID-19 based on symptoms were accepted in high-prevalence areas. The delay between contacts being notified and entering quarantine should be minimal with high levels of public understanding, as should the delay for case isolation. The efficacy of contact tracing (the *y* axis of Fig. 3) is the square of the proportion of the population using the app, multiplied by the probability of the app detecting infectious contacts, multiplied by the fractional reduction in infectiousness resulting from being notified as a contact.

## Ethical considerations

Successful and appropriate use of the app relies on it commanding well-founded public trust and confidence. This applies to the use of the app itself and of the data gathered. There are strong, well-established ethical arguments recognizing the importance of achieving health benefits and avoiding harm. These arguments are particularly strong in the context of an epidemic with the potential for loss of life on the scale possible with COVID-19. Requirements for the intervention to be ethical and capable of commanding the trust of the public are likely to comprise the following: (i) oversight by an inclusive and transparent advisory board, which includes members of the public; (ii) the agreement and publication of ethical principles by which the intervention will be guided; (iii) guarantees of equity of access and treatment; (iv) the use of a transparent and auditable algorithm; (v) integrating evaluation and research in the intervention to inform the effective management of future major outbreaks; (vi) careful oversight of and effective protections around the uses of data; (vii) sharing of knowledge with other countries, especially low- and middle-income countries; and (viii) ensuring that the intervention involves the minimum imposition possible and that decisions in policy and practice are guided by three moral values: equal moral respect, fairness, and the importance of reducing suffering (*27*). It is noteworthy that the algorithmic approach we propose avoids the need for coercive surveillance, because the system can have very large impacts and achieve sustained epidemic suppression even with partial uptake. People should be democratically entitled to decide whether to adopt this platform. The intention is not to impose the technology as a permanent change to society, but we believe that under these pandemic circumstances it is necessary and justified to protect public health.

## Discussion

In this study, we estimated key parameters of the SARS-CoV-2 epidemic, using an analytically solvable model of the exponential phase of spread and of the impact of interventions. Our estimate of R_0_ is lower than many previous published estimates, for example (*12*,***28*, *29*). These studies assumed SARS-like generation times; however, the emerging evidence for shorter generation times for COVID-19 implies a smaller R_0_. This means that a smaller fraction of transmissions need to be blocked for sustained epidemic suppression (R < 1). However, it does not mean that sustained epidemic suppression will be easier to achieve, because each individual’s transmissions will occur in a shorter window of time after infection, and a greater proportion of them will occur before the warning sign of symptoms. Specifically, our approaches suggest that between one-third and one-half of transmissions occur from presymptomatic individuals. This is in line with estimates of 48% of transmission being presymptomatic in Singapore and 62% in Tianjin, China (*30*), and 44% in transmission pairs from various countries (*31*). Our infectiousness model suggests that the total contribution to R_0_ from presymptomatics is 0.9 (CI, 0.2 to 1.1), almost enough to sustain an epidemic on its own. For SARS, the corresponding estimate was almost zero (*9*), immediately telling us that different containment strategies will be needed for COVID-19.

Transmission occurring rapidly and before symptoms, as we have found, implies that the epidemic is highly unlikely to be contained solely by isolating symptomatic individuals. Published models (*9*–*11*, *32*) suggest that in practice, manual contact tracing can only improve on this to a limited extent: It is too slow, and personnel limitations prevent it from being scaled up once the epidemic grows beyond the early phase. Using mobile phones to measure infectious disease contact networks has been proposed previously (*33*–*35*). Considering our quantification of SARS-CoV-2 transmission, we suggest that this approach, with a mobile phone app implementing instantaneous contact tracing, could reduce transmission enough to achieve R < 1 and sustained epidemic suppression, thereby stopping the virus from spreading further. We have developed a web interface to explore the uncertainty in our modeling assumptions (*24*). This will also serve as an ongoing resource as new data become available and as the epidemic evolves.

We included environmentally mediated transmission and transmission from asymptomatic individuals in our general mathematical framework. However, given current data, the relative importance of these transmission routes remains speculative. Cleaning and decontamination are being deployed to varying levels in different settings, and improved estimates of their relative importance would help to inform this as a priority. Asymptomatic infection has been widely reported for COVID-19 [e.g., (*14*)], unlike for SARS, where this was very rare (*36*). We argue that the reports from the early outbreak in Singapore imply that even if asymptomatic infections are common, onward transmission from this state is probably uncommon, because forensic reconstruction of the transmission networks closed down most missing links. There is an important caveat to this: The Singapore outbreak at that stage was small and has not implicated children. There has been widespread speculation that children could be frequent asymptomatic carriers and potential sources of SARS-CoV-2 (*37*, *38*).

We calibrated our estimate of the overall amount of transmission based on the epidemic growth rate observed in China not long after the epidemic started. Growth in Western European countries so far appears to be faster, implying either shorter intervals between individuals becoming infected and transmitting onward, or a higher R_0_. We illustrate the latter effect in figs. S18 and S19. If this is an accurate picture of viral spread in Europe and not an artifact of early growth, epidemic control with only case isolation and quarantining of traced contacts appears implausible in this case, requiring near-universal app usage and near-perfect compliance. The app should be one tool among many general preventive population measures such as physical distancing, enhanced hand and respiratory hygiene, and regular decontamination.

An app-based intervention could be more powerful than our analysis here suggests, however. The renewal equation mathematical framework we use, although well adapted to account for realistic infectiousness dynamics, is not well adapted to account for the benefits of recursion over the transmission network. Once they have been confirmed as cases, individuals identified by tracing can trigger further tracing, as can their contacts, and so on. This effect was not modeled in our analysis here. If testing capacity is limited, individuals who are identified by tracing may be presumed confirmed upon onset of symptoms, because the prior probability of them being positive is higher than for the index case, accelerating the algorithm further without compromising specificity. With fast enough testing, even index cases diagnosed late in infection could be traced recursively to identify recently infected individuals, both before they develop symptoms and before they transmit. Improved sensitivity of testing in early infection could also speed up the algorithm and achieve rapid epidemic control.

The economic and social impact caused by widespread lockdowns is severe. Individuals on low incomes may have limited capacity to remain at home, and support for people in quarantine requires resources. Businesses will lose confidence, causing negative feedback cycles in the economy. Psychological impacts may be lasting. Digital contact tracing could play a critical role in avoiding or leaving lockdown. We have quantified its expected success and laid out a series of requirements for its ethical implementation. The app we propose offers benefits for both society and individuals, reducing the number of cases and also enabling people to continue their lives in an informed, safe, and socially responsible way. It offers the potential to achieve important public benefits while maximizing autonomy. Specific issues exist for groups within the population that may not be amenable to such an approach, and these could be rapidly refined in policy. Essential workers, such as health care workers, may need separate arrangements.

Further modeling is needed to compare the number of people disrupted under different scenarios consistent with sustained epidemic suppression. But a sustained pandemic is not inevitable, nor is a sustained national lockdown. We recommend urgent exploration of means for intelligent physical distancing via digital contact tracing.

## Supplementary Materials

science.sciencemag.org/content/368/6491/eabb6936/suppl/DC1 Materials and Methods Figs. S1 to S21 References (*41*–*45*)
